# Proximal Effects of a Just-in-Time Adaptive Intervention for Smoking Cessation With Wearable Sensors: Microrandomized Trial

**DOI:** 10.2196/55379

**Published:** 2025-03-19

**Authors:** Christine Vinci, Steve K Sutton, Min-Jeong Yang, Sarah R Jones, Santosh Kumar, David W Wetter

**Affiliations:** 1 Moffitt Cancer Center Tampa, FL United States; 2 University of Memphis Memphis, TN United States; 3 University of Utah Salt Lake City, UT United States

**Keywords:** smoking cessation, mindfulness, ecological momentary assessment, micro-randomized trial, Just-in-Time Adaptive Intervention, JITAI, EMA, ecological momentary, smoking, smokers, quitting, cessation, meditation, mind body, sensors, motivational, tobacco, nicotine, NRT, counseling, wearables, abstinence, stress, craving, adaptive intervention, mobile phone

## Abstract

**Background:**

Tobacco use remains the leading preventable cause of morbidity and mortality in the United States. Novel interventions are needed to improve smoking cessation rates. Mindfulness-based interventions (MBIs) for cessation address tobacco use by increasing awareness of the automatic nature of smoking and related behaviors (eg, reactivity to triggers for smoking) from a nonjudgmental stance. Delivering MBIs for smoking cessation via innovative technologies allows for flexibility in the timing of intervention delivery, which has the potential to improve the efficacy of cessation interventions. Research shows MBIs target key mechanisms in the smoking cessation process and can be used to minimize drivers of smoking lapse.

**Objective:**

This single-arm study investigated the impact of mindfulness-based strategies and motivational messages on proximal outcomes, collected via ecological momentary assessment (EMA), relevant to tobacco abstinence via a microrandomized trial. This approach allows for the evaluation of intervention content on proximal outcomes (eg, reduced negative affect) that are thought to impact positive distal outcomes (eg, smoking abstinence).

**Methods:**

All participants were motivated to quit smoking, and the intervention they received included nicotine replacement therapy, brief individual counseling, and a 2-week Just-in-Time Adaptive Intervention (JITAI) with wearable sensors. Throughout the JITAI period, a single strategy was randomly pushed (vs not) multiple times per day through the smartphone application. An EMA next assessed negative affect, positive affect, mindfulness, abstinence self-efficacy, motivation to quit, craving, and smoking motives. The primary analyses evaluated differences in EMA outcomes (proximal) for when a strategy was pushed versus not pushed. Additional analyses evaluated changes in similar outcomes collected from surveys at the baseline and end-of-treatment visits.

**Results:**

Participants (N=38) were 63% (24/38) female, 18% (7/38) Hispanic or Latino, and 29% (11/38) African American. They had an average age of 49 years and smoked an average of 15 (SD 7.9) cigarettes per day. Results indicated that receiving the JITAI significantly reduced proximal negative affect in the second (and final) week of the intervention. Self-reports provided at baseline and end of treatment showed significant decreases in perceived stress, automaticity of smoking and craving, and a significant increase in abstinence self-efficacy. Increases in abstinence self-efficacy significantly predicted abstinence.

**Conclusions:**

To our knowledge, this is the first study to test the proximal impact of a mindfulness-based JITAI on key variables associated with smoking cessation. Our primary finding was that negative affect was lower following the completion of a strategy (vs when no strategy was delivered) in the final week of the JITAI. Among a larger sample size, future research should extend the length of the intervention to further evaluate the impact of the JITAI, as well as include a comparison condition to further evaluate how each component of the intervention uniquely impacts outcomes.

**Trial Registration:**

ClinicalTrials.gov NCT03404596; https://clinicaltrials.gov/study/NCT03404596

## Introduction

Tobacco use remains the leading preventable cause of morbidity and mortality in the United States. Although the prevalence of smoking has decreased dramatically over the past several decades, 12.5% of the US adult population continues to smoke [1]. Novel smoking cessation interventions are needed, and one approach is to target key precipitants of lapse and relapse that may ultimately lead to long-term abstinence. Mindfulness-based interventions (MBIs) for smoking cessation address tobacco use by increasing awareness of the automatic nature of smoking and related behaviors (eg, reactivity to triggers for smoking) from a nonjudgmental stance [2-6]. Formal and informal mindfulness practices are taught and practiced, and individuals are encouraged to implement these practices while quitting smoking. MBIs have been shown to both reduce negative affect and decouple the link between negative affect and craving or smoking behavior [7-13].

Although MBIs have demonstrated efficacy for smoking cessation via traditional in-person delivery [2-4,6], requiring individuals to attend treatment solely in-person can be burdensome and may result in high attrition. As such, various mHealth MBIs delivered via smartphone have been developed, both as module-based apps and apps that adapt to the unique circumstances of the individual [14-16]. For instance, Craving to Quit, a module-based mindfulness smartphone app, showed promise, although there were no significant differences between craving to quit and the comparison condition (inactive attention control) on biochemically-verified abstinence at 6 months (9.8% vs 12.1%) [15]. In our recent publication of a Just-in-Time MBI for smoking cessation delivered via smartphone, results showed high acceptability and moderate feasibility; biochemically-confirmed abstinence was promising with 34% abstinent at the end of treatment and 21% one month later [16]. The primary focus of this study is to examine the proximal impact of this MBI on intermediary variables (eg, negative affect) of tobacco abstinence via a microrandomized trial (MRT), using the same sample of participants as in [16].

MRTs can allow for the evaluation of a Just-in-Time Adaptive Intervention (JITAI) on proximal outcomes (eg, negative affect) that are thought to mediate distal outcomes (eg, smoking abstinence) [17]. In most cases, MRTs aid in the refinement of intervention components. This includes assessment and evaluation of proximal outcomes over time and across situations. Since smoking lapses are very common during a quit attempt [18-22] and most occur within 1 week of quitting [18,21,22], the JITAI tested in this study was designed to address quitting smoking in the first 2 weeks of a quit attempt. This JITAI was created to present an MBI when triggered (1) by momentary negative affect (NA; both high and low) given it is a key precipitant of lapse [23-27] and (2) by smoking behavior itself. Both NA and smoking were designed to be detected in real-time on a smartphone via the artificial intelligence algorithms cStress and puffMarker, respectively, using data captured by the AutoSense wearable wireless sensor suite [28-30] which was worn throughout the study.

As described in more detail below and elsewhere [16], technology-related issues resulted in challenges regarding the detection of high NA and active smoking, despite extensive testing of the app before data collection. Our experience is consistent with recent reports showing the ability to detect rare events using mHealth apps is a challenge [31-33]. Once we determined that high NA and active smoking were not detected as planned, we focused analyses on all other detected moments (low NA and no smoking periods; additional details are provided below).

Individuals who smoked and were treatment-seeking received the JITAI and brief counseling, which were implemented in the first 2 weeks, along with 6 weeks of nicotine replacement therapy (NRT). Mindfulness-based content delivered via the JITAI was the primary treatment element. Data for the proximal effects of the MRT was acquired via ecological momentary assessment (EMA). That is when an eligible moment of NA or smoking was detected from sensor data, that moment was randomized to either deliver a mindfulness or motivational strategy or not. Motivational messages were included due to high desirability in previous mHealth cessation studies [34-36]. An EMA was pushed immediately after the participant completed the strategy or 5 minutes after the strategy was pushed, whichever came first.

This study focuses on the proximal effects of the JITAI on targeted outcomes collected via EMA among adherent participants, previously defined as those who wore the sensors for more than 70% of the time during the 2-week period and completed a majority of the strategies [16]. Our rationale for examining adherent participants is 2-fold. First, this approach ensured that our analysis had EMA data from participants that covered most if not all, days across the 2-week period and for different moments triggered by the JITAI. Second, given this study presents the first test of the JITAI, focusing our analysis among those who received a sufficient dose of the JITAI allows us to determine if a “signal” of the intervention on proximal outcomes is present. That is, if no signal was detected even among those who adhered to the intervention, then no further study of the intervention would be indicated. On the other hand, some signals would suggest that further study of the JITAI is warranted, along with addressing adherence in future studies. We selected the following outcomes based on previous research indicating their importance in the prediction of lapse [8,10,21,24,37-41] and their potential to be impacted by mindfulness [10,42-45]. We hypothesized that following the delivery of a strategy (vs none) participants would report higher positive affect (PA [43,46-48]), mindfulness [15,49-51], abstinence self-efficacy [44,45,52,53], and motivation to quit [54,55], and report lower NA [3,44,48,56-59], craving [9,11,44,51,60,61], and smoking expectancies [49,62,63].

Additional analyses focused on changes from baseline through end of treatment on conceptually similar constructs (affect, mindfulness, abstinence self-efficacy, and smoking motives) using self-report survey data collected at the in-person baseline and end of treatment visits to capture change over the entire intervention. We present results from the entire sample, as well as the subset of participants defined as adherent. In sum, this paper is the first to report the results of a mindfulness-based JITAI on key variables central to smoking cessation.

## Methods

### Overview

We provide here a high-level overview of the study procedure given the multiple study visits and intervention components, with details presented below. After consent, participants were scheduled to attend 5 in-person visits, which consisted of delivering intervention content and study questionnaires [64]. Visit 1 was scheduled 4 days before quit day, visit 2 was on the quit day, visit 3 was 3 days postquit day, visit 4 was 10 days postquit day, and visit 5 was 28 days postquit day. Visits 1-4 included the first 2-week period when participants wore the study equipment, received the JITAI, completed EMAs, and received brief counseling. Visit 4 is referred to as the “end of treatment” throughout the manuscript. Visit 5 only included study questionnaires. Participants received a 6-week supply of NRT to be used at visit 2 (quit day). Refer to Table 1 for the overall study design.

**Table 1:** Study design.

			Visit 1		Visit 2		Visit 3		Visit 4		Visit 5
Day			–4		Day 0 (Quit Day)		+3		+10 (End of treatment)		+28
**Intervention**											
	Brief counseling	✓		✓		✓		✓		N/A^a^	
	JITAI^b^	✓		✓		✓		✓		N/A	
	NRT^c^			✓		✓		✓		N/A	
**Assessments**											
	Questionnaires	✓		✓		✓		✓		✓	
	EMAs^d^	✓		✓		✓		✓		N/A	

^a^Not applicable.

^b^JITAI: Just-in-Time Adaptive Intervention.

^c^NRT: nicotine replacement therapy.

^d^EMA: ecological momentary assessment.

### Participants

Inclusion criteria were (1) age of 18 years or older; (2) having smoked≥ 3 cigarettes per day for the past year; a carbon monoxide (CO) reading of ≥6 ppm; (3) motivated to quit smoking in the next 30 days; (4) a valid home address and functioning phone number; and (5) being able to read, write, and speak English. Exclusion criteria were (1) contraindication for wearing the nicotine patch, (2) psychosis, (3) having an implanted cardiac rhythm device, (4) use of other tobacco products (e-cigarette use okay), (5) pregnancy or lactation, (6) being unable to wear the sensors or provide good readings of physiological data, (7) actively trying to quit smoking, (8) having another member of the household enrolled in the study, and (9) no previous use of a smartphone.

### Measures

#### Overview

Demographics (eg, sex and age) were collected at baseline, and questionnaire data were collected at the 5 in-person visits [64]. EMA data was collected via the study smartphone app during the 2-week JITAI. Tobacco use was self-reported by participants via the Timeline Followback [65,66] and abstinence was biochemically verified by a CO reading of <6 ppm at each visit. The primary tobacco abstinence outcome was a biochemically-confirmed 7-day point prevalence of abstinence at the end of treatment and follow-up, as reported elsewhere [16].

#### The EMA

EMAs were sent to participants about 50% of the time (to reduce burden) after a randomized push or no push of the JITAI. All EMAs presented the same questions, which took 3-5 minutes to complete. EMA variables examined in this analysis included: NA, PA, smoking-related variables (craving, abstinence self-efficacy, motivation to quit, and smoking expectancies), and state mindfulness. All variables were rated on a 5-point Likert scale (1=strongly disagree through 5=strongly agree).

#### Negative Affect and Positive Affect

Each NA and PA item was prefaced with “Right now, I feel…” Responses to 5 items were averaged for NA: anxious, ashamed, guilty, irritable, and restless. Four responses were averaged for PA: active, determined, enthusiastic, and proud.

#### Smoking-Related Variables

Individual items assessed craving (“I have an urge to smoke”), abstinence self-efficacy (“I am confident in my ability to be abstinent from cigarettes”), motivation to quit (“I am very motivated to be abstinent from cigarettes”), and smoking expectancies (“Smoking would improve my mood, be pleasurable, or help me cope with this situation”).

#### State Mindfulness

Each state mindfulness item was prefaced with “When the phone beeped…” The 6 items assessed 3 constructs: attention (“I was doing things automatically, without being aware of what I was doing” and “I found myself doing things without paying attention”), nonjudgment (“I was telling myself that I shouldn’t be thinking the way I’m thinking” and “I was thinking that some of my emotions are bad or inappropriate and I shouldn’t feel them”), and decentering (“I was experiencing my thoughts more as events in my mind than a direct reflection of the way things really are” and “I was experiencing my changing thoughts and feelings as separate from myself”).

#### Questionnaires

The Positive Affect and Negative Affect Scale (PANAS) is a self-report measure that assesses PANAS-PA and PANAS-NA [67]. The Perceived Stress Scale (PSS) measures the degree to which participants find their lives to be stressful [68,69]. The Five Facet Mindfulness Questionnaire (FFMQ) assesses 5 components of mindfulness including observing, describing, awareness, nonjudgment, and nonreactivity [70]. The Self-Efficacy Scale – Smoking (SES) determines an individual’s level of confidence for not smoking in positive or social situations, negative affect situations, and out of habit [71]. Finally, 2 subscales from the Brief Wisconsin Inventory of Smoking Dependence Motives were analyzed to assess smoking motives related to automaticity and craving [72]. The automaticity subscale was chosen due to its conceptual overlap with mindfulness (ie, automaticity should theoretically reduce overtime via mindfulness) [73,74] and craving was chosen due to its association with lapse [24,75,76].

### Procedures

Participants were recruited from the community via advertisements and contacted the study team to complete an initial phone screen, where they were provided with general information about the study. Those eligible and interested were invited to an in-person orientation session where more detail about the study was provided and eligibility screening was finalized (ie, CO, pregnancy test, assessment of psychosis symptoms via the Mini-International Neuropsychiatric Interview [77]). Those still eligible and interested were scheduled for 5 in-person visits.

At visit 1 (4 days prequit), participants completed the informed consent, baseline questionnaires, and CO measurement. They were taught how to wear and use the equipment. The study smartphone was provided with instructions on its use and for strategy and EMA completion. Participants then completed brief counseling (refer to below). Visits 2 (quit day), 3 (3 days postquit day), and 4 (10 days postquit day) included the administration of visit questionnaires, CO measurement, an equipment check, and brief counseling. Use of NRT began at visit 2 and continued for 6 weeks; dosing was based on participants’ reported cigarettes smoked per day. At visit 4 (end of treatment), all equipment was returned. At visit 5 (28 days postquit day), participants completed questionnaires and CO measurements, and additional smoking cessation resources were provided.

### Ethical Considerations

The study protocol was approved by the institution’s institutional review board (MCC19002). Informed consent was obtained from all participants and included the option to opt out of the study at any time. The data collected and results presented in this manuscript were covered within the scope of the informed consent document. Data was deidentified. Participants were compensated at each visit for completing measures (US $10 at orientation, US $30 at visits 1-3; US $50 at visits 4 and 5). Bonus compensation was earned by completion of the EMAs. If participants wore the equipment for at least 60% of the time since the last phone prompt, they received US $1.25 for each EMA completed. If equipment had not been worn at least 60% of the time, they received US $.50 for completing each EMA.

### AutoSense

AutoSense [28-30] is a wearable sensor suite that consists of a chest strap with 2 electrodes and 2 wrist sensors worn by participants during the study. A smartphone with the study app collected all data from AutoSense sensors wirelessly. NA was detected from respiration and electrocardiogram data, and the cStress algorithm was used to categorize a given moment as low versus high NA [29]. Smoking status was detected from a combination of respiration and hand-to-mouth movement from wrist sensors. The PuffMarker algorithm determined whether a given moment was smoking versus no smoking [30]. A more detailed description of the cStress and Puffmarker algorithms can be found elsewhere [16,29,30,64].

#### MRT Design

Specific moments during the day were designed to be randomized to either push intervention content to a participant, or not, and the decision point for randomization was based on smoking status, NA, and availability (which were continuously monitored via AutoSense and the study smartphone). Each day was separated into six 2-hour blocks for the delivery of mindfulness or motivational strategies (ie, the JITAI). For low NA and no smoking, the decision to randomize was based on a randomly chosen time at the beginning of the 2-hour block. If the participant was available and experiencing low NA (or was not smoking) based on data collected from the equipment, randomization occurred. If unavailable (eg, driving), a new time was chosen within the block, and randomization was again attempted at that time. This continued until the end of the block. Within a 2-hour block, randomization was limited to once for low NA and once for no smoking to limit the burden. Participants could accept, delay, or ignore the strategy, and after completion, participants rated the strategy. An EMA then appeared about 50% of the time following randomization of a strategy and participants had the option to complete, delay, or ignore.

#### Intervention Content

The JITAI consisted of mindfulness or motivational strategies sent to participants each day of the 2-week period when wearing the equipment. Mindfulness strategies (n=76) were designed to prompt individuals to engage in mindfulness skills at that moment and fell into one of 5 topic areas: breath, thoughts, sensations, acceptance or nonjudgment, and craving. The first 4 topic areas were chosen as they represent the broad areas of focus that mindfulness-based meditations and exercises often fall under [78], while also providing a practical framework to develop statements that individuals can understand and implement with ease in the present moment. Craving was added as a category to provide mindfulness strategies that were tailored to the experience of quitting smoking. Examples included “Turn your attention to your breathing. Notice where you feel your breathing most in your body,” “Our thoughts change each moment. Notice how quickly your thoughts may shift around over the next several moments,” and “Notice how the clouds in the sky are constantly moving and never stay in the same place. Cravings are similar – they come and go.” Motivational messages (n=40) were designed to support motivation to quit smoking, as previous research has identified these types of messages as appealing and helpful for those making a quit attempt [34-36]. Examples included “Every cigarette you don’t smoke is money saved. Great job!” and “You got this. Keep moving towards your goal of being tobacco free.” Participants could also access the mindfulness strategies on demand (ie, when not prompted) through a button within the app. Another on-demand button addressed 13 frequently asked questions about mindfulness (eg, Is mindfulness the same thing as meditation?).

Brief individually-delivered counseling was consistent with the Treating Tobacco Dependence Guidelines (eg, removing smoking paraphernalia, and social support) [79] and lasted about 20-30 minutes at each visit. Visit 1 provided participants with the National Cancer Institute’s Clearing the Air booklet and a single-page handout on mindfulness. During visits 1-3, participants also listened to a 10-minute audio recording of mindfulness meditation (either breath meditation or urge surfing), followed by a brief discussion about the meditation with the study counselor.

#### Analytic Plan

Descriptive statistics were used to summarize participant characteristics, and to review EMA item responses to identify outliers and patterns of responding that would suggest invalid data (eg, straight-lining). Participants who wore the sensors for at least 8 days and completed at least 60% of the delivered strategies were previously identified as adherent [16]. The primary analyses evaluated the JITAI impact on proximal outcomes among this subset who engaged with the JITAI as intended on self-reported NA, PA, smoking-related variables (craving, abstinence self-efficacy, motivation to quit, and smoking expectancies), and state mindfulness using generalized estimating equations (GEE). The primary model included strategy push (vs no-push), condition (low NA vs not smoking), day of intervention (1-14), and hour of the day (0-23). Three intervention periods were analyzed separately: (1) prequit period (4 days) evaluated differences while still smoking, (2) postquit period (10 days) evaluated differences while attempting to quit, and (3) the final 7 days evaluated differences after 1 week of experience with the intervention and practicing mindfulness.

Unexpected technology-related issues resulted in the detection of very few moments of high NA (the algorithm was too stringent) and smoking (there was a mismatch of orientation parameters in the wrist sensors) among participants [16]. Specifically, 6 EMAs were completed following the detection of high NA and 5 following smoking (less than 2% of all EMAs completed). Thus, we did not include these few moments in the analysis because of the technology-related concerns already noted, as well as the inability of these moments to reasonably represent the intended condition.

Additional analyses focused on the impact of the entire intervention by applying paired sample *t* tests to evaluate changes in PANAS-NA, PANAS-PA, perceived stress, mindfulness, abstinence self-efficacy, and smoking motives (automaticity and craving) at baseline and end of treatment. These analyses were performed for the entire sample (n=38) as well as the subset of participants considered adherent (n=16). Changes in these measures were also evaluated as predictors of abstinence at the end of treatment using simple logistic regression.

## Results

### Overview

Of the 43 who consented, four dropped out of the study after visit 1, and one was unable to wear the sensors due to being ill. Of the remaining 38 participants, 24 wore the sensors for at least 8 of the 14 days. Of those 24 participants, 16 participants completed at least 60% of the strategies sent. Table 2 presents participant characteristics for those who completed visit 4 (n=38), as well as adherent participants (n=16). There were no significant group differences between those who were adherent and those who were not (n=22).

There were 704 EMAs completed by the 16 adherent participants. This included 178 EMAs during prequit, 526 during postquit, and 374 during the final 7 days. There were no response patterns observed that necessitated the removal of data before analysis (eg, straight-lining).

**Table 2:** Participant characteristics.

Variable						Adherent (n=16)		All (n=38)				*P* value^a^	
Age (years), mean (SD)						49.6 (13.5)		49.4 (13.5)				.96	
Gender: women, n (%)						10 (62.5)		24 (63.2)				.94	
Ethnicity: Hispanic or Latinx						3 (18.8%)		7 (18.4%)				.98	
**Race, n (%)**													.79
	Black or African American				5 (31.3)		11 (28.9)						
	White				11 (68.8)		27 (71.1)						
**Relationship status, n (%)**													.94
		Married or living together as married			6 (37.5)		13 (34.2)						
		Single			6 (37.5)		15 (39.5)						
		Other			4 (25)		10 (26.3)						
Education: high school diploma, General Education Development, or less, n (%)				5 (31.3)						11 (28.9)		.72	
Annual household income <US $30,000, n (%)				9 (56.3)						22 (57.9)		.38	
Heaviness of Smoking Index^b^, mean (SD)				3.0 (1.4)						2.8 (1.3)		.55	
Cigarettes per day, mean (SD)			16.6 (8.5)						15.4 (7.9)			.43	

^a^*P* value for statistical test comparing the 16 participants categorized as adherent versus the 22 participants considered not adherent. The *t* test was used for continuous variables and the chi-square test was used for categorical variables.

^b^Heaviness of Smoking Index consisted of 2 items: time to first cigarette and cigarettes per day.

### JITAI Proximal Outcomes

Table 3 presents the results from the GEE model of the effect of pushing a strategy on each proximal outcome for each of the three JITAI periods. Multimedia Appendix 1 presents results for all model components. NA was significantly lower after a strategy was pushed (estimated mean [EM] 1.98) versus not pushed (EM 2.07) during the final week of the intervention. No significant differences were observed during the prequit or postquit periods between when the intervention was pushed versus not. PA did not exhibit any statistically significant differences.

**Table 3:** Results by intervention period among adherent participants (n=16).

Outcome	Intervention period, B^a^ (*P* value)		
	Prequit	Postquit	Final week
Negative affect	0.061 (.34)	–0.041 (.20)	–0.090 (.01)^b^
Positive affect	–0.095 (.09)	0.010 (.80)	0.032 (.47)
Motivation	0.063 (.50)	–0.015 (.71)	0.052 (.33)
Abstinence self-efficacy	0.005 (.97)	–0.001 (.98)	0.088 (.07)
Expectancies	–0.011 (.90)	–0.062 (.28)	–0.123 (.051)
Craving	0.055 (.60)	0.017 (.82)	–0.022 (.83)
Attention	–0.083 (.41)	0.014 (.82)	–0.042 (.60)
Non-judgement	0.032 (.51)	0.032 (.35)	0.067 (.10)
Decentering	–0.097 (.32)	0.063 (.10)	0.096 (.08)

^a^B: unstandardized beta.

^b^Statistically significant differences (α=.014).

Refer to Table S1 in Multimedia Appendix 1 for a presentation of all model components.

There were no statistically significant differences for any of the 4 smoking-related variables. However, during the final week, differences trended in the expected direction for expectancies (EMs are 2.41 and 2.53, respectively) and for abstinence self-efficacy (EMs are 3.88 and 3.79), although not significant.

There were no statistically significant differences for any of the 3 mindfulness-related variables. However, during the final week, differences for push versus no-push for decentering trended in the expected direction, albeit not significant (EMs are 2.13 and 2.04).

### Baseline to End of Treatment Changes

Table 4 presents descriptive statistics and results of paired *t* tests. Among adherent participants, a significant increase in PANAS-PA (*P*=.03) and a significant decrease in perceived stress were observed (*P*=.02). The nonjudgment subscale of the FFMQ increased (*P*=.03). All subscales of the Abstinence Self-Efficacy Scale significantly increased: Negative/Affective, Positive/Social, Habit/Addictive, and total score (all *P*s<.001). Regarding smoking motives, both automaticity (*P*<.01) and craving (*P*=.02) significantly decreased.

Among the entire sample, a significant decrease in perceived stress was observed (*P*<.01). All subscales of the Abstinence Self-Efficacy significantly increased: negative or affective, positive or social, habit or addictive, and total score (all *P*s<.001). Both automaticity (*P*<.01) and craving subscales (*P*<.01) significantly decreased. No other significant changes were observed.

In total, 13 of 38 (34%) participants were biochemically confirmed abstinent at the end of treatment [16]. Increases in abstinence self-efficacy (total score) significantly predicted abstinence odd ratio (odds ratio [OR] 2.57, 95% CI 1.21-5.44; *P*=.01). Of all other measures, only PANAS-NA showed a trend in the expected direction as related to abstinence (OR 0.87, 95% CI 0.75-1.02; *P*=.08).

**Table 4:** Change in affect, mindfulness, and smoking-related variables from baseline to end of treatment.

		Adherent participants (n=16)						Full sample (n=38)						
		Baseline, mean (SD)	End of treatment, mean (SD)	Test statistic				Baseline, mean (SD)		End of treatment, mean (SD)		Test statistic		
				*t* test (*df*)	*P* values	Cohen *d*					*t* test (*df*)		*P* values	Cohen *d*
**Positive and Negative Affect Scale**														
	Negative affect	16.44 (4.76)	15.75 (4.65)	0.49 (15)	.63	0.12	19.47 (8.74)		18.84 (8.52)		0.74 (37)		.46	0.12
	Positive affect	30.38 (8.02)	35.25 (7.93)	–2.33 (15)	.03	0.58	32.55 (9.56)		34.82 (8.74)		–1.81 (37)		.08	0.29
	Perceived stress scale	7.38 (2.58)	5.44 (3.08)	2.55 (15)	.02	0.64	7.37 (3.78)		6.11 (3.73)		2.93 (37)		.01	0.48
**Five Facet Mindfulness Questionnaire**														
	Observation	25.73 (8.03)	26.67 (5.73)	–0.60 (14)	.56	0.16	27.32 (6.99)		27.14 (5.76)		0.23 (36)		.82	0.04
	Describing	29.93 (5.87)	29.40 (6.19)	0.34 (14)	.74	0.09	29.73 (6.53)		29.89 (6.20)		–0.18 (36)		.86	0.03
	Attention or awareness	30.20 (8.55)	29.07 (7.41)	0.60 (14)	.56	0.15	30.11 (7.39)		29.76 (7.01)		0.37 (36)		.72	0.06
	Nonjudgment	30.60 (6.85)	33.20 (5.66)	–2.35 (14)	.03	0.61	28.38 (7.85)		30.16 (8.45)		–2.03 (36)		.05	0.33
	Nonreactivity	20.33 (6.01)	19.87 (5.51)	0.30 (14)	.77	0.08	21.35 (6.40)		20.84 (5.67)		0.53 (36)		.60	0.09
**Self-Efficacy Scale – Smoking**														
	Negative or effective	1.73 (0.85)	3.19 (0.86)	–5.50 (15)	<.001	1.38	1.70 (0.76)		2.96 (1.20)		–7.14 (37)		<.001	1.16
	Positive or social	1.56 (0.76)	3.33 (1.02)	–5.21 (15)	<.001	1.30	1.81 (0.84)		3.30 (1.20)		–7.10 (37)		<.001	1.15
	Habit or addictive	1.96 (0.97)	3.67 (0.92)	–5.33 (15)	<.001	1.33	2.14 (1.01)		3.60 (1.21)		–7.14 (37)		<.001	1.16
	Total	1.75 (0.78)	3.40 (0.80)	–5.82 (15)	<.001	1.46	1.88 (0.78)		3.28 (1.11)		–7.62 (37)		<.001	1.24
**Brief Wisconsin Inventory of Smoking Dependence Motives**														
	Automaticity	4.23 (1.63)	2.73 (2.05)	3.32 (15)	.01	0.83	4.32 (1.86)		3.16 (2.14)		3.71 (37)		.01	0.60
	Craving	4.70 (1.38)	3.34 (1.72)	2.70 (15)	.02	0.67	4.65 (1.32)		3.42 (1.85)		4.08 (37)		<.001	0.66

## Discussion

### Overview

This study evaluated the momentary impact of mindfulness-based JITAI on proximal outcomes, as well as the broader impact of the entire intervention on conceptually similar constructs assessed via self-report surveys at baseline and end of treatment. The JITAI strategies significantly reduced proximal NA in the second (ie, final) week. The intervention also produced hypothesized changes from baseline to end of treatment with decreases in perceived stress, automaticity of smoking, and craving, and increases in abstinence self-efficacy. Additional increases in PANAS-PA and nonjudgment were observed among adherent participants.

### Principal Findings

To our knowledge, this is the first study to test the proximal impact of mindfulness-based JITAI on key variables associated with smoking cessation. Our primary finding was that NA reduced following the completion of a strategy (vs when no strategy was delivered) in the final week of the JITAI. Both mindfulness and motivational strategies were sent, and although the majority were mindfulness, these results suggest that such a combination has a positive effect on NA in the context of quitting smoking. As reported previously [16], participants reported that the just-in-time aspect was one of their favorite parts of the intervention, as well as the ability of the strategies to help them self-regulate, stay present, and manage cravings. Future research should evaluate whether sending a strategy at different moments in time (eg, high vs low NA) results in different proximal effects.

Other proximal outcomes (ie, expectancies that smoking would improve mood, abstinence self-efficacy, decentering) exhibited differences that trended in the expected direction, albeit nonsignificant. Together with the significant reduction in NA, these findings suggest that there may be a cumulative impact of receiving these strategies that results in observable differences in the final week. In other words, increased experience and practice with the strategies result in a more substantial effect later in the intervention period. Future testing of this JITAI should examine this hypothesis by extending the delivery of the JITAI over additional weeks and among a larger sample, as well as including a comparison condition to determine if these findings are unique to this JITAI. For instance, Garrison et al [15] found that craving to quit resulted in increased mindfulness, although this occurred for both the mindfulness and control condition.

This JITAI was delivered in the context of other treatment components, including brief in-person counseling and NRT. When examining how the entire intervention impacted conceptually similar outcomes over time, we observed reductions in perceived stress and smoking motives of automaticity and craving, along with increases in abstinence self-efficacy; additionally, among adherent participants, significant increases in PANAS-PA and the mindfulness construct of nonjudging were observed. Finally, increases in abstinence self-efficacy were significantly predictive of abstinence. These results are consistent with the broader literature on mindfulness interventions for smoking cessation [10,42-45]. For those receiving the JITAI as intended (ie, adherent participants), not only were the additional benefits of increased PA and nonjudgment found, but the effect sizes on these outcomes were larger for this subset of participants. This finding may not be particularly surprising, as we would expect that those who were more adherent would also obtain greater benefits. Given the numerous components of this intervention and the potential to vary the intensity of each, future research may consider an optimization trial (eg, factorial experiment) to examine the unique benefits of each component as related to treatment efficacy and cost-effectiveness.

Results from this study point to the importance of addressing engagement in the context of mHealth interventions. For instance, our previous paper found that adherent participants reported significantly more experience with wearable sensors at baseline, as compared to nonadherent participants [16]. This suggests future research should directly address adherence by (1) allowing participants a few days to gain experience with the equipment before starting the study and/or (2) using less burdensome equipment. Fortunately, rapid advances in technology are making equipment more feasible to wear (eg, wristbands only). Extant research indicates that adding features such as gamification and progress tracking increases adherence and engagement [35,80] (which were not present in this version of the app). Future research should incorporate such features to hopefully increase adherence. Relatedly, although biochemically confirmed abstinence was not statistically different [16], these numbers trended toward higher abstinence among those who were more engaged with the intervention, further underscoring the importance of addressing engagement. Finally, advances in artificial intelligence could address engagement by leveraging patterns of when participants do engage with the app (eg, sending more vs less prompts), current activities (eg, driving), and general schedule (eg, at work so unavailable).

A brief discussion of nonsignificant findings is warranted despite the smaller-than-intended sample size and low statistical power. First, the JITAI intervention did not impact proximal outcomes during prequit nor during the first several days post quitting. One potential explanation is that participants needed more time to practice the intervention strategies. Second, even in the final week of the JITAI, changes were not observed in some variables (eg, aspects of mindfulness, PA, craving). This may be due to limited power to detect changes, that the intervention content (mindfulness and motivational strategies) did not have an immediate impact on these variables, or even that negative affect (relative to the other variables we tested) may be more sensitive to change via brief mindfulness strategies. Future research is needed to investigate these possibilities. Third, we observed changes from baseline to end of treatment on certain variables (eg, positive affect) that we did not see immediately impacted by the strategies. The reverse is also true – NA was immediately impacted following the completion of a strategy within the JITAI, whereas PANAS-NA assessed at baseline and end of treatment did not change. There are various explanations for these conflicting findings including that the variables were measured in different ways (ie, the survey measure items were different than the EMA measures); that one set of measures captured the impact of the entire intervention across all components over a long period of time whereas the other set captured the immediate impact of a single intervention strategy; and the expectation was that the JITAI would have a small effect on proximal outcomes, although extending the JITAI for another week may have strengthened these effect sizes. Fourth, we did not observe significant changes on all subscales of the FFMQ, which may be due to the brief period of the intervention, the intervention itself, or other measurement-related issues (eg, demand characteristics, specific constructs measured by the FFMQ [81]).

Limitations first include that there was no control condition, as the primary goal was to evaluate the proximal impact of receiving (vs not) a strategy. Thus, it is unclear whether the results may simply reflect the natural history of these outcomes over the first 2 weeks of any cessation intervention or even just the impact of brief counseling and NRT, particularly for changes observed from baseline through the end of treatment. Without a comparison condition sending non-mindfulness or motivational strategies, it is also not possible to conclude that the NA reductions were due to mindfulness or motivational strategies (vs some other type of strategy). Results should be interpreted with caution until future studies can be conducted with a control condition. Second, given the small sample size, additional research is needed to confirm our findings with a larger sample. Third, although our JITAI was deliberately designed to be implemented within the first 2 weeks of quitting smoking to address the period when most smoking lapses occur [18,22,27], we are limited in our ability to draw conclusions on the impact of the intervention beyond this short timeframe. Fourth, given technology issues, we are unable to know with certainty that the low NA and no smoking moments did not include moments of high NA and smoking. As such, we focused our conclusions solely on whether a strategy was pushed or not, although future research using appropriate sensor detection could further investigate such important distinctions.

### Conclusion

In sum, this paper reports on the first investigation of a mindfulness-based JITAI on proximal outcomes related to smoking cessation. Key takeaways include the fact that proximal NA was affected by strategies in the final week of the JITAI with other variables trending in the expected direction. One recommendation is to extend the intervention so that participants have more time to learn and practice the strategies. Whereas there were observed changes from baseline to end of treatment for many variables, there needs to be a comparison condition to better understand these changes and evaluate how different components of the intervention (JITAI, in-person counseling, and NRT) impact outcomes. Finally, given that participants who were adherent to wearing the equipment and engaging with the intervention content exhibited larger differences in outcomes, it would be beneficial to address engagement with this type of intervention in future studies.

## Appendix

Multimedia Appendix 1 Results for all model components for each outcome by each intervention period.

Multimedia Appendix 2 CONSORT-eHEALTH checklist (V 1.6.1).

## Abbreviations

CO: carbon monoxide
EM: estimated mean
EMA: ecological momentary assessment
FFMQ: Five Facet Mindfulness Questionnaire
GEE: generalized estimating equations
JITAI: Just-in-Time Adaptive Intervention
MBI: mindfulness-based intervention
MRT: microrandomized trial
NA: negative affect
NRT: nicotine replacement therapy
OR: odds ratio
PA: positive affect
PANAS: Positive Affect and Negative Affect Scale
PSS: Perceived Stress Scale
SES: Self-Efficacy Scale – Smoking
