# Supplementary material for: Proximal Effects of a Just-in-Time Adaptive Intervention for Smoking Cessation With Wearable Sensors: Microrandomized Trial
Source: JMIR Mhealth Uhealth. 2025 Mar 19;13:e55379. doi: 10.2196/55379 (PMC11966069; doi:10.2196/55379)
Supplement: Multimedia Appendix 1 [file mhealth_v13i1e55379_app1.docx]

Table S1.

Results for all model components for each outcome by each intervention period

|  | **Intervention Period** | | |
| --- | --- | --- | --- |
| **Outcome/Predictors** | **Pre-quit** | **Post-quit** | **Final Week** |
|  | B (p-value) | B (p-value) | B (p-value) |
| Negative Affect |  |  |  |
| MBI Pushed  (versus Not Pushed) | 0.061 (.336) | -0.041 (.200) | **-0.090 (.014)** |
| Study day | -0.025 (.654) | -0.008 (.668) | -0.045 (.084) |
| Hour of day | 0.016 (.081) | -0.001 (.831) | 0.005 (.457) |
| Low Stress  (versus Not Smoking) | -0.007 (.842) | -0.024 (.472) | -0.028 (.539) |
| Positive Affect |  |  |  |
| MBI Pushed  (versus Not Pushed) | -0.095 (.091) | 0.010 (.804) | 0.032 (.469) |
| Study day | 0.052 (.306) | -0.006 (.714) | -0.018 (.607) |
| Hour of day | **-0.029 (.028)** | -0.008 (.421) | -0.015 (.173) |
| Low Stress  (versus Not Smoking) | 0.029 (.626) | -0.008 (.763) | 0.001 (.986) |
| Motivation |  |  |  |
| MBI Pushed  (versus Not Pushed) | 0.063 (.500) | -0.015 (.708) | 0.052 (.326) |
| Study day | 0.098 (.135) | 0.012 (.635) | 0.001 (.969) |
| Hour of day | 0.015 (.333) | -0.001 (.912) | 0.005 (.596) |
| Low Stress  (versus Not Smoking) | 0.102 (.264) | -0.066 (.216) | -0.038 (.515) |
| Abstinence Self-efficacy |  |  |  |
| MBI Pushed  (versus Not Pushed) | 0.005 (.967) | -0.001 (.979) | 0.088 (.068) |
| Study day | 0.014 (.852) | 0.036 (.240) | 0.020 (.628) |
| Hour of day | -0.006 (.809) | -0.003 (.736) | -0.002 (.890) |
| Low Stress  (versus Not Smoking) | 0.011 (.893) | -0.030 (.447) | -0.007(.871) |

Continued on next page.

Table S1 (continued).

|  | **Intervention Period** | | |
| --- | --- | --- | --- |
| **Outcome/Predictors** | **Pre-quit** | **Post-quit** | **Final Week** |
|  | B (p-value) | B (p-value) | B (p-value) |
| Expectancies |  |  |  |
| MBI Pushed  (versus Not Pushed) | -0.011 (.897) | -0.062 (.276) | -0.123 (.051) |
| Study day | -0.085 (.196) | -0.035 (.287) | 0.036 (.426) |
| Hour of day | -0.030 (.146) | -0.012 (.282) | 0.001 (.967) |
| Low Stress  (versus Not Smoking) | -0.064 (.522) | 0.020 (.747) | 0.010 (.869) |
| Craving |  |  |  |
| MBI Pushed  (versus Not Pushed) | 0.055 (.603) | 0.017 (.822) | -0.022 (.825) |
| Study day | -0.094 (.336) | -0.052 (.105) | -0.020 (.641) |
| Hour of day | **-0.056 (.042)** | -0.020 (.133) | 0.004 (.830) |
| Low Stress  (versus Not Smoking) | 0.007 (.942) | -0.011 (.870) | -0.074 (.241) |
| Attention |  |  |  |
| MBI Pushed  (versus Not Pushed) | -0.083 (.409) | 0.014 (.823) | -0.042 (.600) |
| Study day | **-0.131 (.039)** | 0.041 (.193) | -0.003 (.930) |
| Hour of day | -0.019 (.277) | 0.018 (.250) | 0.031 (.093) |
| Low Stress  (versus Not Smoking) | -0.136 (.415) | -0.008 (.888) | -0.043 (.633) |
| Non-judgement |  |  |  |
| MBI Pushed  (versus Not Pushed) | 0.032 (.509) | 0.032 (.346) | 0.067 (.097) |
| Study day | 0.080 (.119) | -0.007 (.633) | **-0.061 (.026)** |
| Hour of day | 0.017 (.145) | 0.016 (.143) | 0.014 (.353) |
| Low Stress  (versus Not Smoking) | -0.066 (.074) | 0.007 (.836) | -0.025 (.607) |

Continued on next page.

Table S1 (continued).

|  | **Intervention Period** | | |
| --- | --- | --- | --- |
| **Outcome/Predictors** | **Pre-quit** | **Post-quit** | **Final Week** |
|  | B (p-value) | B (p-value) | B (p-value) |
| Decentering |  |  |  |
| MBI Pushed  (versus Not Pushed) | -0.097 (.320) | 0.063 (.101) | 0.096 (.079) |
| Study day | -0.026 (.485) | **0.066 (.047)** | **0.082 (.043)** |
| Hour of day | 0.004 (.695) | 0.004 (.427) | 0.008 (.253) |
| Low Stress  (versus Not Smoking) | -0.001 (.979) | 0.070 (.064) | 0.072 (.104) |

Note. Statistically significant differences are in **bold** (α=.05). B = unstandardized beta.
